# Bearing-Fault-Feature Enhancement and Diagnosis Based on Coarse-Grained Lattice Features

**DOI:** 10.3390/s24113540

**Published:** 2024-05-30

**Authors:** Xiaoyu Li, Baozhu Jia, Zhiqiang Liao, Xin Wang

**Affiliations:** 1 Naval Architecture and Shipping College, Guangdong Ocean University, Zhanjiang 524088, China; yuxiaoenn@163.com (X.L.); jiabzh@gdou.edu.cn (B.J.); wx2112005027@126.com (X.W.); 2 Technical Research Center for Ship Intelligence and Safety Engineering of Guangdong Province, Zhanjiang 524088, China; 3 Guangdong Provincial Key Laboratory of Intelligent Equipment for South China Sea Marine Ranching, Guangdong Ocean University, Zhanjiang 524088, China

**Keywords:** rolling bearings, fault diagnosis, coarse-grained lattice features, Swin Transformer, feature enhancement

## Abstract

In view of the frequent failures occurring in rolling bearings, the strong background noise present in signals, weak features, and difficulties associated with extracting fault characteristics, a method of enhancing and diagnosing rolling bearing faults based on coarse-grained lattice features (CGLFs) is proposed. First, the vibrational signals of bearings are subjected to adaptive filtering to eliminate background noise. Second, frequency-domain transformation is performed, and a coarse-grained approach is used to continuously segment the spectrum. Within each segment, amplitude-enhancement operations are executed, transforming the data into a CGLF graph that enhances fault characteristics. This graph is then fed into a Swin Transformer-based pattern-recognition network. Third and finally, a high-precision fault diagnosis model is constructed using fully connected layers and Softmax, enabling the diagnosis of bearing faults. The fault recognition accuracy reaches 98.30% and 98.50% with public datasets and laboratory data, respectively, thereby validating the feasibility and effectiveness of the proposed method. This research offers an efficient and feasible fault diagnosis approach for rolling bearings.

## 1. Introduction

Rolling bearings serve as crucial supporting components for rotary equipment, playing a pivotal role in ensuring its normal operation. Bearing failures, accounting for approximately 41% of failure rates, are among the most frequent issues encountered in rotary equipment. Such failures can incapacitate the equipment, resulting in significant economic losses and potentially even casualties. Consequently, monitoring equipment status and diagnosing faults are paramount [1,2]. The working environment of bearings is typically complex and variable, and they are influenced by factors such as background noise and alternating load impacts. Accordingly, the vibration signals collected often exhibit subtle fault characteristics amidst numerous forms of interference. This necessitates the enhancement of weak fault characteristics to effectively improve fault recognition accuracy [3].

The issue of enhancing weak fault features in rotating equipment’s rolling bearings has been studied by numerous scholars. Techniques such as time-domain, frequency-domain, and time–frequency analysis, as well as signal–modal decomposition and filtering methods, are extensively utilized for fault feature enhancement [4]. Early bearing-fault analyses are relatively straightforward, relying on time-domain statistical analysis, including root-mean-square values, moments, spectral envelope analysis, and cepstral analysis [5,6,7,8,9]. However, these methods often prove ineffective owing to noise and interference from various sources [10]. Some classical time–frequency signal enhancement methods possess unresolved inherent flaws, such as the limited adaptability of the short-time Fourier transform (STFT), which is unable to fully satisfy the Heisenberg uncertainty principle [11]. Other challenges include selecting wavelet transform basis functions [12] and modal confusion issues with empirical mode decomposition (EMD) [13]. Accordingly, researchers have developed variants of EMD, including local mean decomposition [14,15], intrinsic time-scale decomposition [16], and local feature-scale decomposition [17]. However, the application of these variants and the original EMD method may give rise to similar drawbacks, such as boundary effects, modal mixing, a lack of a mathematical theory, and computational inefficiency [18]. Filtering is an effective method for noise reduction and fault feature enhancement. For instance, Yu et al. [19] propose using the harmonic significance index to quantify fault information in frequency bands, thereby determining bandpass filter parameters. Ma et al. [20] also describe the use of frequency-slice wavelet transform to select frequency bands for early fault transient signals. Given that fault signal resonance frequencies and bandwidths can be arbitrary, these methods require the manual adjustment of bandwidths, making the selection of appropriate fault-filtering frequency bands crucial [21]. Spectral kurtosis (SK) can represent the smoothness of a signal waveform and reflect impact fault features. Wang et al. [22] further discuss the use of kurtosis and gear-meshing index to extract resonant frequency bands related to bearing and gear faults. Wang et al. [23] introduce a maximum-kurtosis spectral-entropy deconvolution-fault feature-enhancement algorithm, which has been successfully applied for gearbox fault diagnosis. Therefore, this paper adopts an adaptive SK for extracting fault-related central frequency bands.

Pattern recognition is a critical step in equipment diagnostics. Deep learning theory, with its ability to stack multiple layers and multi-structured information representation layers, demonstrates hierarchical information representation capabilities in data modeling processes. This has become a research hotspot in the field of fault diagnosis [24]. Jiachi et al. implement a Deep Transfer Convolutional Neural Network (DTCNN) to achieve an accurate estimation of lithium-ion battery capacity [25]. Hoang et al. [26] apply convolutional neural networks (CNNs) for pattern recognition, but the limited receptive field of CNNs’ convolutional kernels leads to suboptimal diagnostic results. A vision transformer (ViT)-based local perception transformer fault diagnosis model is also proposed in the literature [27]. It overcomes the aforementioned CNN issues but neglects the correlation between local features. To address these issues, this paper uses a pattern-recognition method based on Swin Transformer. Swin Transformer integrates the hierarchical architecture of CNNs with the global self-attention mechanism of ViT, dividing the image into multiple windows to compute the self-attention within each window, thereby reducing computational load. It also uses a shifting window operation to enable information exchange between windows, thereby capturing global image information and effectively achieving multi-scale feature extraction and recognition [28].

To address the aforementioned situation, this paper proposes a feature enhancement and diagnosis method based on coarse-grained lattice features (CGLFs) which leverages the advantages of Swin Transformer in feature extraction and recognition. Vibration signals from bearings under various states are collected and labeled with state tags and then divided into training, validation, and test sets. The original signals undergo adaptive bandpass filtering to eliminate background noise, followed by fast Fourier transformation (FFT) for frequency-domain transformation to obtain spectra. The spectra are then continuously segmented using a coarse-grained method, with amplitude enhancement operations performed within each segment, transforming them into CGLF graphs with enhanced fault characteristics. These graphs are inputted into the Swin Transformer pattern-recognition network. Finally, a high-precision fault diagnosis model is established through fully connected layers and Softmax, facilitating bearing-fault diagnosis. The proposed method is validated on public datasets and laboratory data, confirming its feasibility and effectiveness.

## 2. Theoretical Background

### 2.1. Adaptive Bandpass Filtering

When local damage occurs in a bearing, high-frequency vibrations are excited owing to the impacts between components during loaded operation [29]. Filtering methods are effective for studying impact frequencies. Traditional bandpass filters require engineers to repeatedly adjust filter parameters, affecting diagnostic efficiency [30]. Accordingly, this paper adopts an adaptive filtering method based on SK.

Wang et al. [31] define SK based on Wold–Crammer decomposition, which describes any random non-stationary process as the output of a causal, linear, and time-varying system, i.e.,

(1) $$x(n)=\int_{-\frac{1}{2}}^{\frac{1}{2}}H(n,f)e^{j2\pi /n}dZ_{x}(f)$$

In the equation, *dZ_x_*(*f*) is the standard orthogonal spectral increment, and *H*(*n,f*) is the complex envelope of *x*(*n*) at frequency f [32].

In SK, *K_x_*(*f*) is defined as the fourth-order normalized cumulant:

(2) $$K_{x}(f)=\frac{\langle {\left|H(n,f)\right|}^{4}\rangle }{{\langle {\left|H(n,f)\right|}^{2}\rangle }^{2}}-2$$

The equation shows that the kurtosis value of a stationary process is a constant function of frequency, and the kurtosis value of a Gaussian process is zero. The frequency band dominated by transient impact signals has a larger SK, whereas the frequency band dominated by stationary Gaussian noise has a smaller one. Therefore, the frequency band of transient impact signals can be identified by the magnitude of the kurtosis value [33]. Consequently, the frequency band of the bandpass filter is determined by the size of the kurtosis value and changes with it, thereby achieving adaptive determination of the central frequency band.

### 2.2. CGLF Principle

CGLF is a feature-enhancement fusion method based on spectral analysis. It can effectively extract fault features under strong background noise. It is simple in structure and has high computational efficiency. First, FFT is used to transform the signal from the time domain to the frequency domain to obtain the spectrum. 

Step 1: A coarse-grained sampling coefficient is set, and the spectrum is segmented into continuous *j* segments, with amplitude and enhancement operations performed within each segment. The specific calculation process is as follows:

(3) j=fix(r/d)

(4) $$F_{cn}(j)=sum(F_{c}((j-1)\times d+1:j\times d))$$

Here, *d* is the coarse-grained sampling coefficient, *r* is the spectral width, *fix*() represents a function that performs downward rounding, and *F_cn_* represents the corresponding amplitude value.

Step 2: Based on the number of new features, we set the feature value range (*S* = *j*) and adjust the magnitude of each feature accordingly within the range (*S*).

(5) $$F_{cm}(j)=fix(\frac{(F_{cn}(j)-F_{cnmin})\times S}{F_{cnmax}-F_{cnmin}})$$

Here, *F_cm_*() denotes the final feature values, *F_cnmin_* is the minimum feature value, and *F_cnmax_* is the maximum feature value.

### 2.3. Evaluation Metrics for Diagnostic Models

The model performance is evaluated using four parameters: Accuracy (*Acc*), Precision (*P*), Recall (*R*)*,* and *F1* Score. Accuracy represents the ratio of correctly predicted samples to the total number of samples. Precision indicates the proportion of true positive samples among those predicted as positive by the model, reflecting the accuracy of the predictions. Recall represents the proportion of true positive samples correctly identified by the model, indicating the model’s ability to recognize positive samples. The *F1* Score is the harmonic mean of Precision and Recall, used for a comprehensive evaluation of the model’s performance, especially in cases of imbalanced data.

(6) $$Acc=\frac{TP+TN}{TP+TN+FP+FN}\times 100\%$$

(7) $$P=\frac{TP}{TP+FP}\times 100\%$$

(8) $$R=\frac{TP}{TP+FN}\times 100\%$$

(9) $$F1=2\frac{P*R}{P+R}\times 100\%$$

In Equations (6)–(9), *TP* represents the number of true positive samples, *TN* denotes the number of true negative samples, *FP* indicates the number of false positive samples, and *FN* signifies the number of false negative samples.

### 2.4. Swin Transformer

**Figure 1:**
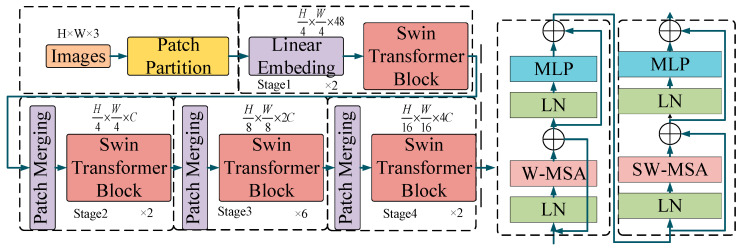
The architecture of Swin Transformer.

Swin Transformer (Figure 1), improved by incorporating the ViT and CNN models, shows potential beyond classic CNNs in image processing and pattern-recognition tasks [34,35]. Swin Transformer blocks comprise a Window Multi-head Self-Attention (W-MSA) module and a Shifted Windows Multi-Head Self-Attention (SW-MSA) module, followed by two Multilayer Perceptrons (MLPs). They are connected through a GELU activation function to enhance the nonlinear capabilities of the entire network. Before W-MSA, SW-MSA, and MLP, a normalization layer (LN) exists, and each module is followed by a residual connection to improve the fitting ability of the entire network structure. The flow of the entire Swin Transformer algorithm is as follows: A three-channel RGB image signal is used as input, and the original image is cut into non-overlapping patches through a patch partition operation, resulting in 4 × 4 patches. After flattening the patches, features with the shape (H/4, W/4, 48) are obtained. These features are then inputted into a linear embedding layer for dimensionality reduction, yielding a feature block with the shape (H/4, W/4, C). This feature block serves as input to the Swin Transformer module for feature extraction. After completing a Stage operation, the image size is halved, and the channel number is doubled through a patch-merging operation. The output features after each Stage are (H/8, W/8, 2C), (H/16, W/16, 4C), and (H/32, W/32, 8C), respectively. Finally, image feature recognition classification is achieved through a fully connected layer and Softmax.

## 3. Fault Diagnosis Process

**Figure 2:**
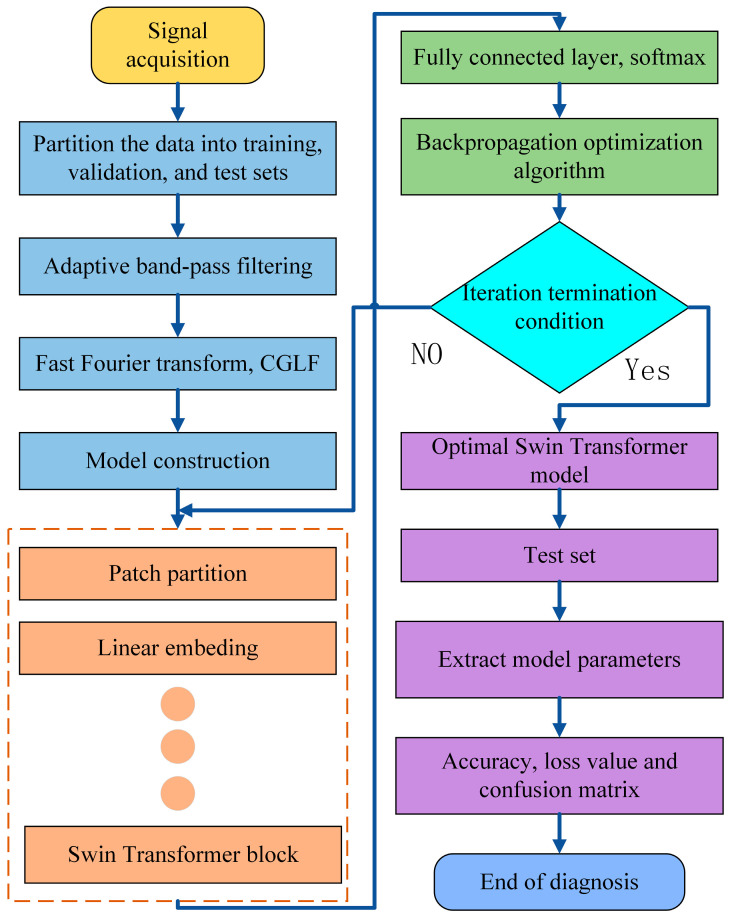
Flowchart of rolling-bearing = fault diagnosis.

The fault diagnosis process proposed in this paper based on CGLF is described as follows: Vibration signals of bearings under different states are collected, labeled with status tags, and divided into training, validation, and test sets. The vibration signals are subjected to adaptive bandpass filtering, followed by frequency-domain transformation to obtain the spectrum. The spectrum is then segmented continuously using the coarse-graining method to transform it into a CGLF graph with enhanced fault characteristics. This graph is inputted into the Swin Transformer pattern-recognition network. Finally, a high-precision fault diagnosis model is established through fully connected layers and Softmax, achieving fault diagnosis of the bearings. The flowchart of the process is shown in Figure 2. The pseudocode for calculating the CGLFs is shown in Algorithm 1.
**Algorithm 1.** Pseudocode for CGLFInput: The collected vibration signal data Output: Plots of CGLF 1: Load the data 2: Extract data from the struct 3: Set sampling_rate 4: Compute the FFT of the extracted data 5: Get the length of the data 6: Compute frequency bins 7: Select frequency indices within the specified range 8: Extract corresponding FFT values 9: Extract corresponding frequencies 10: Compute absolute values of the selected FFT values 11: Set coarse sampling factor: d 12: Compute spectrum length: r 13: j = floor(r/d) 14: For e from 1 to j: 15:  av = (e − 1) × d + 1 16:  bv = e × d 17:  xa = abs_fft(a:b) 18:  k = sum(xa) 19:  kv (e) = k 20:  y = min(kv) 21:  z = max(kv) 22:  cg = floor((k − y)/(z − y) × j) 23:  cg_v (e) = cg 24: end for 25: For e from 1 to j: 26:  Scatter plot cg_v for the current segment 27:  hold on 28: end for

The major steps are as follows:

Step 1: Vibration signals of the rolling bearings are collected, and fixed-length time series under different states are extracted. Then, they are numbered and divided into training, validation, and test sets according to proportion.

Step 2: The fault feature frequency band is determined through an adaptive bandpass filter, and then FFT transformation is performed. We set a coarse-graining coefficient and use the coarse-graining method to continuously segment the spectrum, converting it into a CGLF graph with enhanced fault characteristics.

Step 3: A Swin Transformer network is constructed, and the relevant parameters are set. After inputting the training set into the network in batches for hyperparameter training, the trained network model is saved.

Step 4: The test set is inputted to validate the trained model and identify the bearing status.

## 4. Experimental Verification and Analysis

### 4.1. Public Dataset Validation

**Figure 3:**
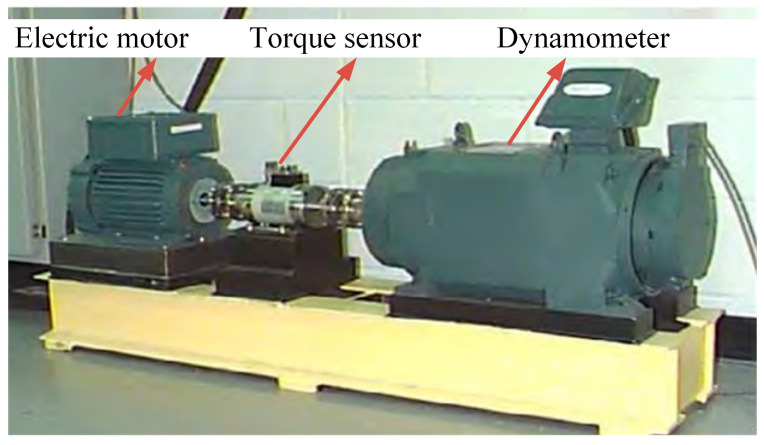
CWRU bearing-fault experiment platform.

The feasibility of the method proposed in this paper is verified using experimental data from the bearing-fault experiment platform of the Electrical Laboratory at Case Western Reserve University (CWRU). The experimental platform comprises a motor, torque sensor, dynamometer, and electronic controller (Figure 3). The drive-end bearing model is a 6205-2RS deep-groove ball bearing, and the bearing vibration signals are collected using an accelerometer. The bearing faults are induced by electro-spark single-point damage. The experiment is conducted with a rotation speed of 1750 rpm, a load of 2 HP, a sampling frequency of 12 kHz, a sampling time of 0.682 s, and 8192 sampling points. A total of 10 types of data are selected for four states: rolling element faults, outer ring faults, inner ring faults, and normal bearings. The specific experimental data are presented in Table 1, including data for fault sizes of 7 mils, 14 mils, and 21 mils.

**Figure 4:**
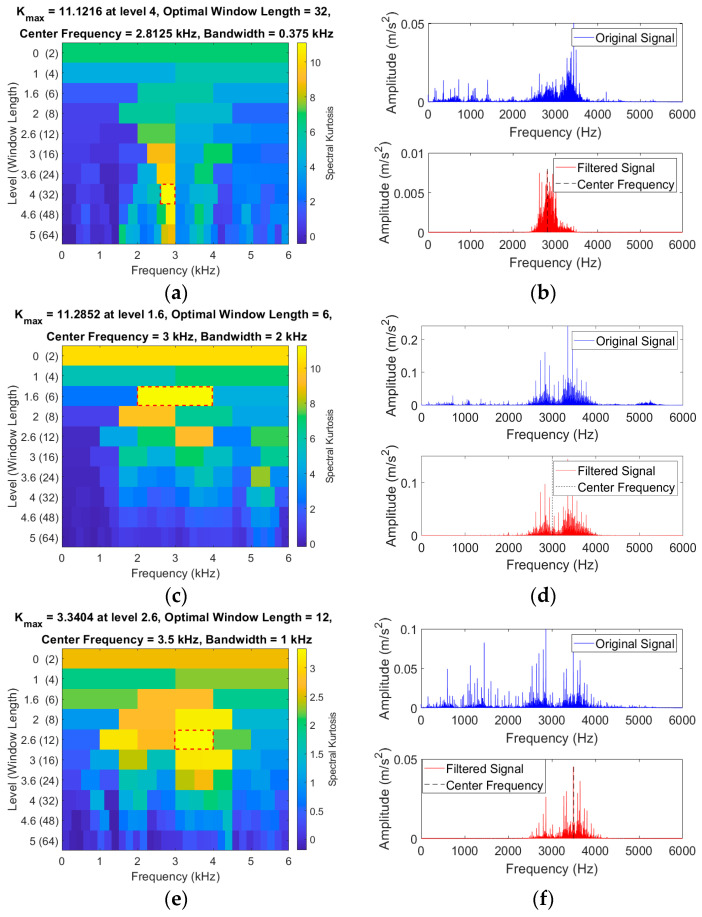

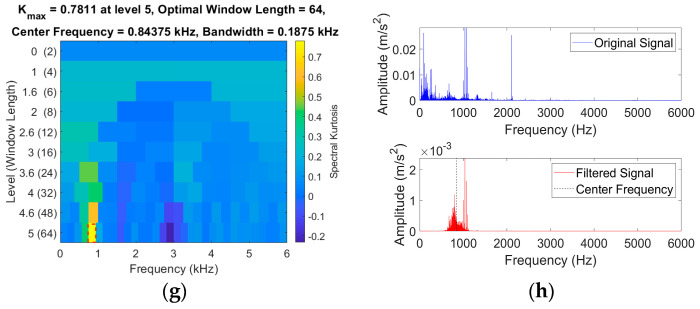
SK graphs of bearings in four conditions and spectral diagrams before and after filtering. (**a**) SK graph (Ball fault). (**b**) Frequency domain (Before and after ball fault filtering). (**c**) SK graph (Outer ring fault). (**d**) Frequency domain (Before and after outer fault filtering). (**e**) SK graph (Inner ring fault). (**f**) Frequency domain (Before and after inner fault filtering). (**g**) SK graph (Normal bearing). (**h**) Frequency domain (Before and after normal bearing filtering).

The adaptive SK method is used to process the CWRU laboratory’s fault-bearing data, and the results are shown in Figure 4, which shows the SK graphs of the vibration signals for the rolling element faults, outer ring and inner ring faults, and normal bearings, as well as the corresponding original signals and filtered spectra.

**Figure 5:**
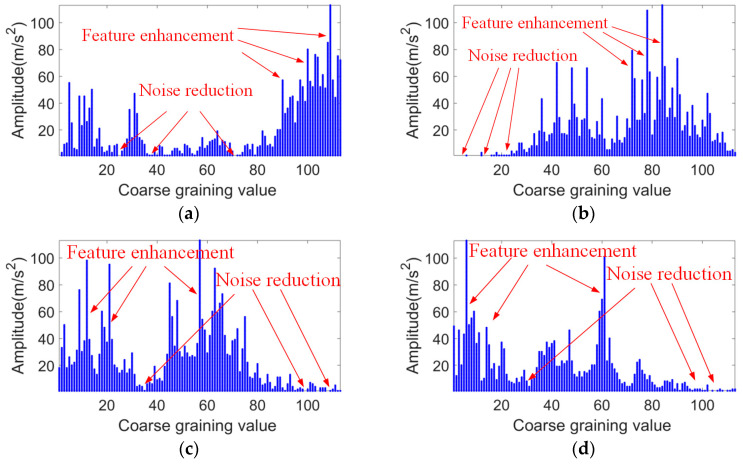
CGLF graph for four bearing states. (**a**) Ball fault. (**b**) Outer ring fault. (**c**) Inner ring fault. (**d**) Normal bearing.

Figure 4 further shows that the highest kurtosis values of the vibration signals for the rolling element faults, outer ring faults, inner ring faults, and normal bearings are located at the center frequencies of 2.81, 3.0, 3.5, and 0.84 kHz, respectively. The bandwidths where the potential fault information is located are 0.37, 2.0, 1.0, and 0.19 kHz, respectively. Considering the above, a signal segment with a bandwidth of 2 kHz centered at the center frequency is selected for analysis and processing. A coarse-graining sampling coefficient of 12 is set, and the coarse-grained lattice feature values within the bandwidth are calculated, resulting in 113 feature values. They are then transformed into a CGLF graph (Figure 5).

**Figure 6:**
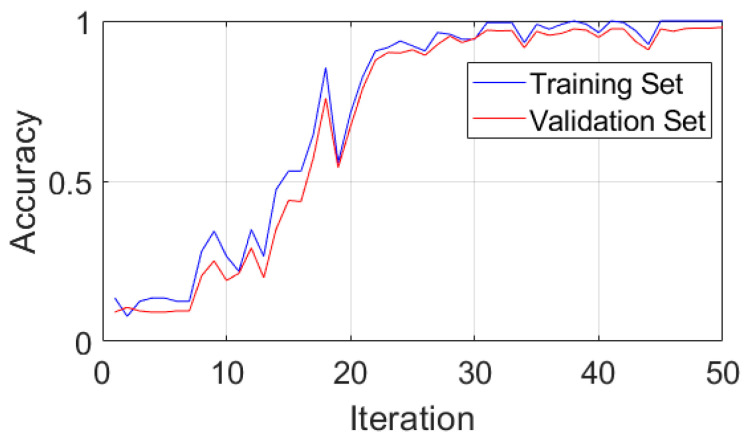
Accuracy curves for the training and validation sets.

**Figure 7:**
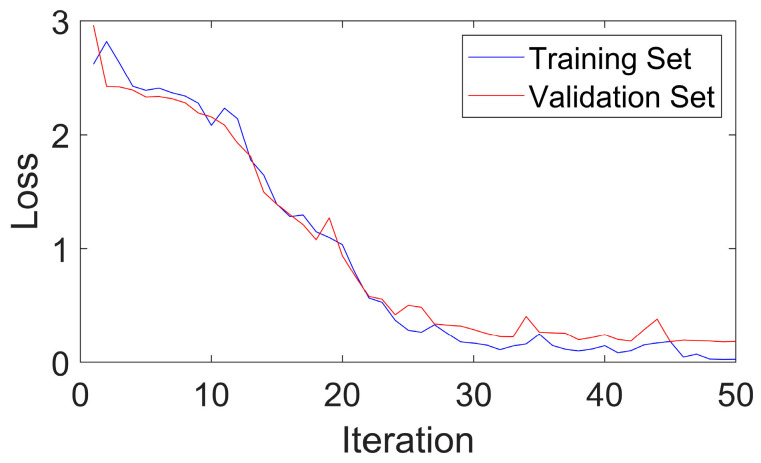
Loss curves of the training and validation sets for the public dataset.

**Figure 8:**
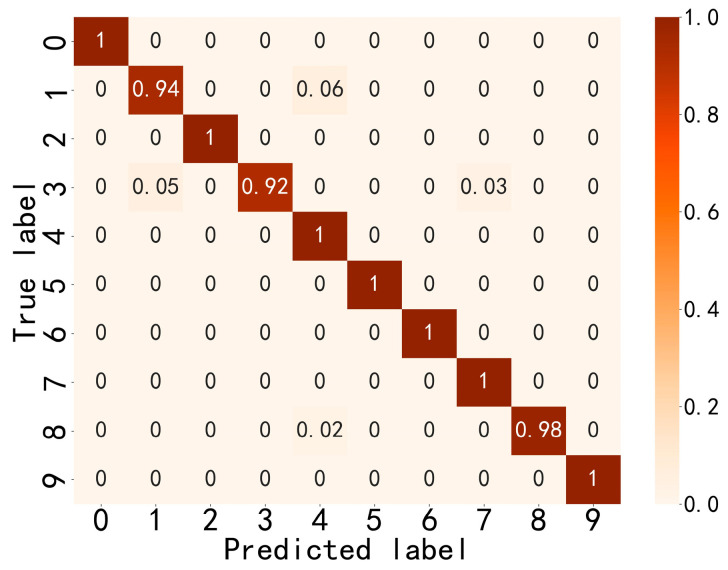
Confusion matrix for the test sets.

Figure 5 shows that CGLF can reduce the impact of noise and enhance the fault impact components, demonstrating a significant data feature enhancement and fusion effect. The Swin Transformer network is constructed, and the input network parameters are set as follows: the input image is adjusted to 224 × 224, the batch processing size is 16, the learning rate is 1 × 10^−3^, the weight decay is 1 × 10^−5^, the number of iterations is 50, the number of classification categories is 10, the optimizer is stochastic gradient descent, and the loss function is the cross-entropy loss function. The experimental results are extracted from the training logs and analyzed visually, as shown in Figure 6, Figure 7 and Figure 8.

Figure 6 and Figure 7 indicate that the method proposed in this paper has good fault-state-identification performance. The training curve accuracy gradually increases, reaching 100% around the 45th iteration, and the validation set accuracy reaches 97.99%. The optimal model is loaded, and the test set accuracy reaches 98.30%. In terms of stability, the accuracy and loss curves are generally stable, indicating superior stability and small training fluctuations. An analysis of Figure 8 reveals that the model proposed in this paper also demonstrates satisfactory performance in distinguishing between similar, easily confused types and faults of varying degrees. In general, the method proposed in this paper exhibits excellent performance in terms of identification accuracy, convergence, stability, and classification of faults with different severities, thereby validating the feasibility and effectiveness of the proposed approach.

### 4.2. Experimental Test Validation

**Figure 9:**
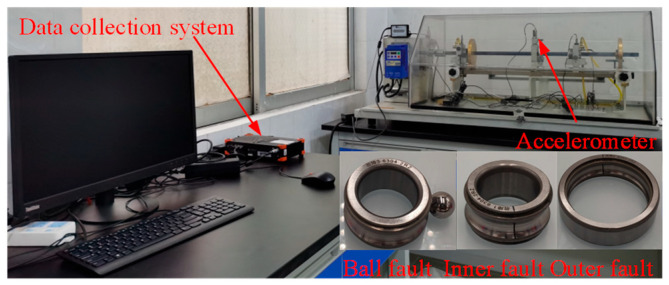
Bearing-fault test bench.

The practical performance of the model built in this paper is further validated on a bearing-fault test bench (Figure 9). The fault bearing used in this test bench is a 6304 deep-groove ball bearing (parameters shown in Table 2), with a rotation speed of 1200 rpm, a sampling frequency of 16,384 Hz, and a 0 HP load condition. Vibration signals are obtained through a ZD-518 radial acceleration sensor (100 mv/g), and the test data include 100 samples each for outer ring faults, inner ring faults, rolling element faults, composite faults, and normal conditions. Each sample has 8192 sampling points, divided into training, validation, and test sets in a 3:1:1 ratio (Table 3).

**Figure 10:**
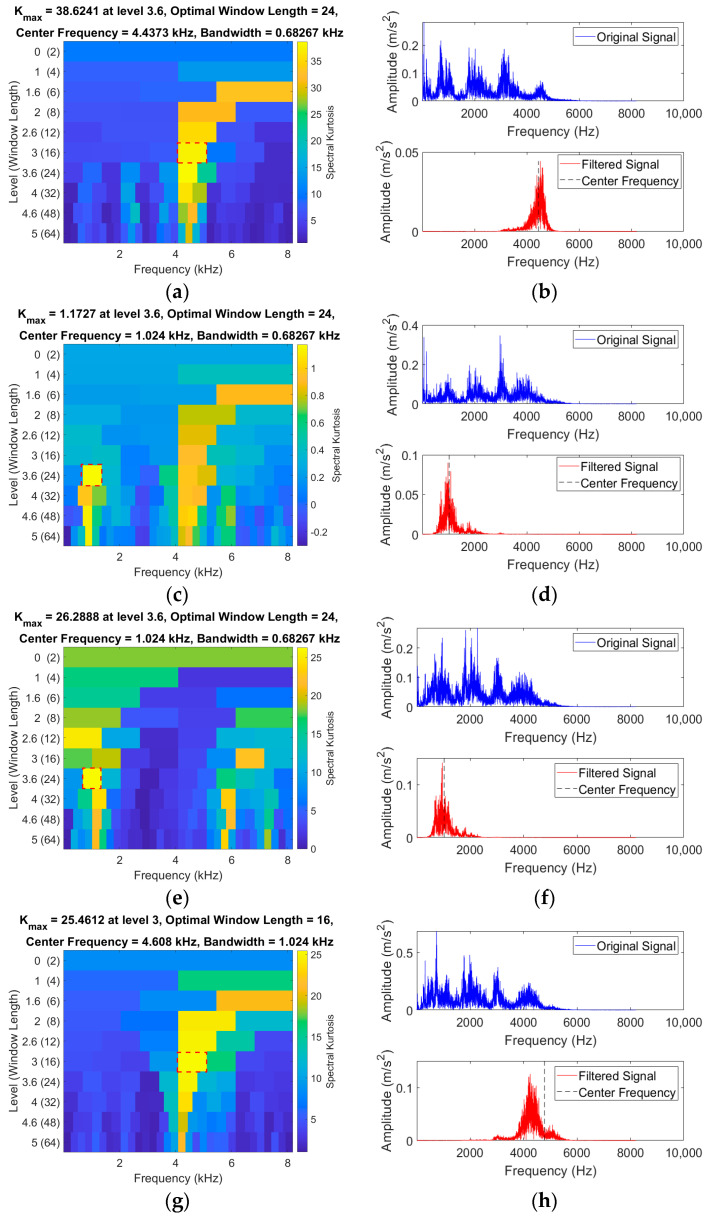

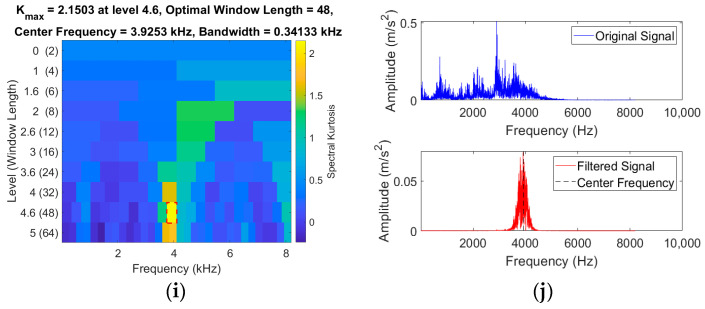
SK graphs of bearings in five conditions and spectral diagrams before and after filtering. (**a**) SK graph (Ball fault). (**b**) Frequency domain (Before and after ball fault filtering). (**c**) SK graph (Outer ring fault). (**d**) Frequency domain (Before and after outer fault filtering). (**e**) SK graph (Inner ring fault). (**f**) Frequency domain (Before and after inner fault filtering). (**g**) SK graph (composite fault). (**h**) Frequency domain (Before and after composite fault filtering). (**i**) SK graph (Normal bearing). (**j**) Frequency domain (Before and after normal bearing filtering).

The adaptive SK method is used to process the laboratory fault-bearing data, and the results are shown in Figure 10. Figure 10 shows the SK graphs of the vibration signals for the rolling element, outer ring, inner ring, and composite faults and normal bearings, as well as the corresponding original signals and filtered spectra.

**Figure 11:**
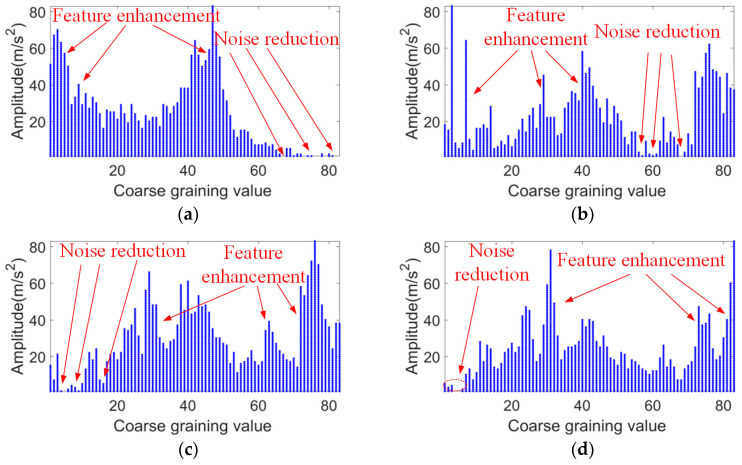

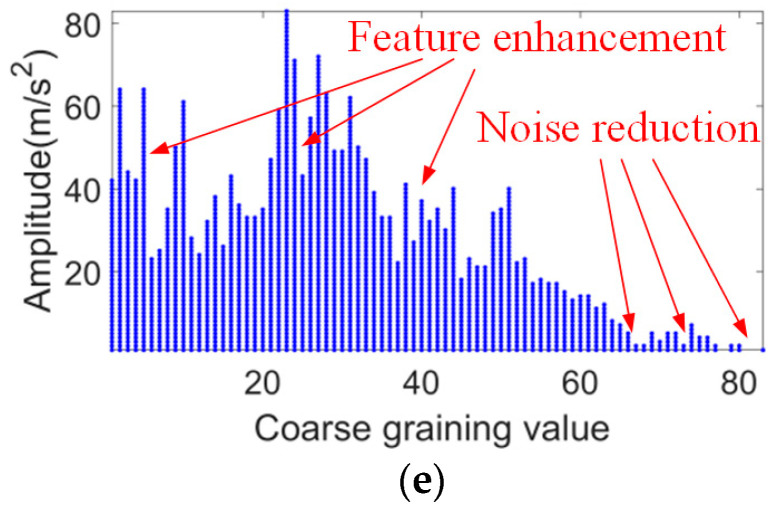
CGLF graph for five bearing states. (**a**) Ball fault. (**b**) Outer race fault. (**c**) Inner race fault. (**d**) Composite fault. (**e**) Normal bearing.

Analyzing the SK graphs of rolling element faults, outer ring faults, inner ring faults, composite faults, and normal bearing vibration signals in Figure 10, the center frequencies are found to be 4.43, 1.024, 1.02, 4.60, and 3.92 kHz, respectively. The fault impact signals are concentrated in bandwidths of 0.68, 0.68, 0.68, 1.02, and 0.34 kHz, respectively. Considering the above, a signal segment with a bandwidth of 2 kHz centered at the center frequency is selected for analysis and processing. A coarse-graining sampling coefficient of 12 is set, and the coarse-grained lattice feature values within the bandwidth are calculated. This results in 83 feature values, which are transformed into a CGLF graph (Figure 11).

**Figure 12:**
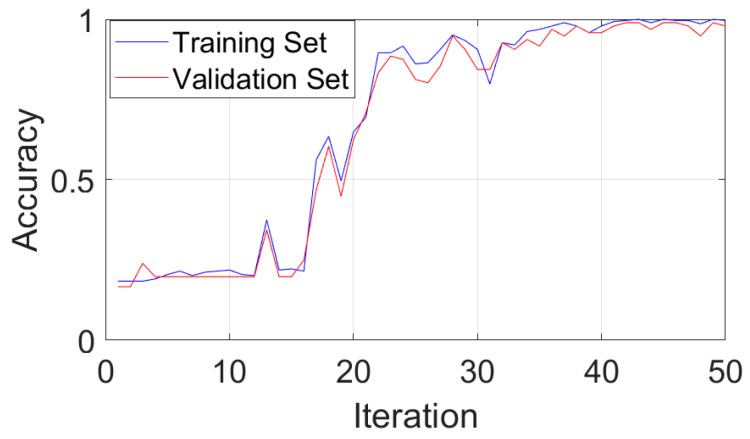
Accuracy curves for the training and validation set.

**Figure 13:**
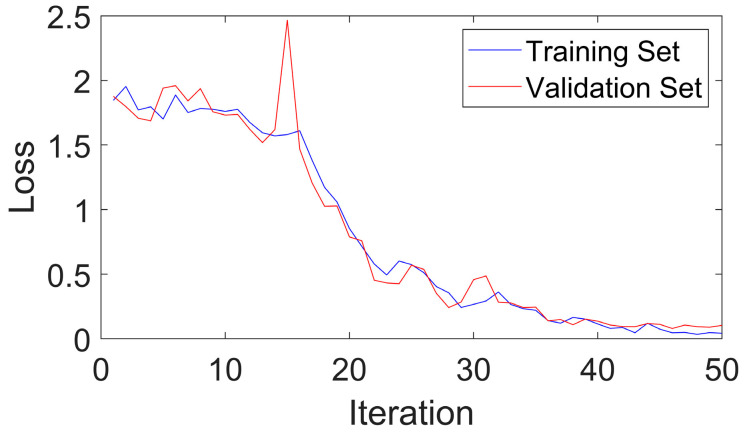
Loss curves of the training and validation sets for Bearing-fault test bench data.

**Figure 14:**
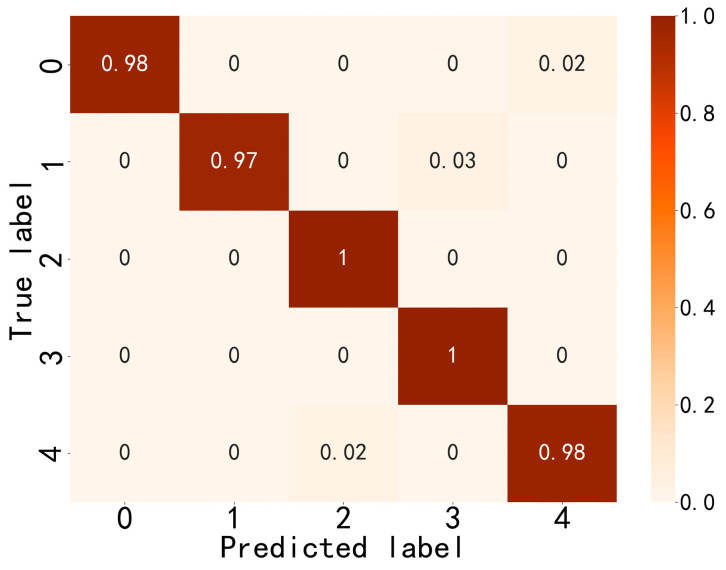
Confusion matrix for the test set.

Figure 11 shows that CGLF can reduce the impact of noise and enhance the fault impact components, demonstrating a significant data feature enhancement and fusion effect. The Swin Transformer network is initialized, and the input network parameters are set as follows: the input image is adjusted to 224 × 224, the batch processing size is 16, the learning rate is 1 × 10^−3^, the weight decay is 1 × 10^−5^, the number of iterations is 50, the number of classification categories is 5, the optimizer is stochastic gradient descent, and the loss function is the cross-entropy loss function. The experimental results are extracted from the training logs and analyzed visually, as shown in Figure 12, Figure 13 and Figure 14.

Figure 12 indicates that the method proposed in this paper has good fault-state-identification performance. The training set accuracy steadily increases, reaching 100% at around the 42nd iteration. The validation set accuracy reaches 97.91%. The optimal model is loaded, and the test set accuracy reaches 98.50%, further proving the good stability and scalability of the proposed method. Figure 13 shows that the loss values of the training and validation set steadily decrease and converge, indicating the good generalization ability of the proposed method. An analysis of Figure 14 shows that the proposed method also performs well in distinguishing between similar faults and faults with different degrees of severity. In summary, the method exhibits good stability and scalability, excellent generalization performance, and superior performance in classifying similar and different degrees of faults, confirming its feasibility and effectiveness.

### 4.3. Comparative Experiment

To further validate the diagnostic effectiveness of the proposed method, it is compared and analyzed with some existing research results. (1) The S-CNN method first obtains the time–frequency diagram of the original data of the bearings through S-transform and then extracts the features through CNN for fault classification [36]. (2) The STFT-sparse autoencoder (SAE) method obtains the time–frequency diagram of the original vibration signal through STFT, uses a stacked SAE network to extract fault features, and realizes fault classification through softmax regression [37]. (3) The original voltage signals are proposed to be converted into grayscale images using empirical wavelet transform (EWT), and the proposed deep CNN model is applied to classify the EWT grayscale images for fault diagnosis [38]. (4) Wavelet transform is used to decompose and reconstruct the signals, which are then used as inputs for the back propagation neural network (BPNN) for decision making and classification [39]. To ensure the comparability of the results, the above algorithms use the public bearing dataset from CWRU and the laboratory-collected dataset, with specific results shown in Table 4.

### 4.4. Analysis of Test Results

The experimental results on the CWRU public dataset and laboratory data indicate that the proposed method performs well in terms of Accuracy, Precision, Recall, and F1 Score, surpassing other methods, as shown in Table 4. Specifically, on the public dataset, the proposed method achieved an Accuracy of 98.30%, a Precision of 99.0%, an F1 Score of 98.7%, and a Recall of 98.4%. On the laboratory dataset, it achieved an Accuracy of 98.50%, a Precision of 99.2%, an F1 Score of 98.9%, and a Recall of 98.6%. These comparative experiments further demonstrate that the proposed method effectively enhances the integration of fault features, improves the distinction between fault types, and provides better generalization performance and stability, thereby achieving efficient intelligent fault diagnosis for rolling bearings.

## 5. Conclusions

This paper proposes a feature enhancement and diagnosis method based on CGLF and validates it using the CWRU public dataset and laboratory data. The results indicate that the proposed method can effectively achieve intelligent fault diagnosis for rolling bearings. The method is computationally simple, simplifying the feature extraction process and reducing computational complexity through spectrum segmentation and amplitude enhancement. Adaptive bandpass filtering automatically determines the frequency band range, reducing human intervention and randomness, thereby improving the reliability of the results. The method demonstrates superior generalization and stability across different datasets, excelling at classifying similar faults and faults with varying degrees of severity, confirming its feasibility and effectiveness in practical applications. In summary, the proposed method is computationally simple, is low in randomness, and exhibits good generalization and stability, providing significant theoretical and practical value for the safe and stable operation of rolling bearings.

## Figures and Tables

**Table 1:** CWRU bearing-fault experiment platform data.

Sample Type	Fault Diameter/Mils	Label	Training Set	Validation Set	Test Set
Normal bearing	0	0	60	20	20
Inner ring fault	7/14/21	1/2/3	60/60/60	20/20/20	20/20/20
Ball fault	7/14/21	4/5/6	60/60/60	20/20/20	20/20/20
Outer ring fault	7/14/21	7/8/9	60/60/60	20/20/20	20/20/20

**Table 2:** Bearing parameters.

Component	Value
Bore diameter	0.787 in
Outside diameter	2.047 in
Width	0.591 in

**Table 3:** Bearing-fault test bench data.

Sample Type	Training/Validation/Test Set	Label
Normal bearing	60/20/20	0
Inner ring fault	60/20/20	1
Ball fault	60/20/20	2
Outer ring fault	60/20/20	3
Combination fault	60/20/20	4

**Table 4:** Comparison of different diagnostic methods.

Model	Dataset Types	Accuracy	Precision	F1 Score	Recall
S-CNN	CWRU dataset	92.10	89.83	86.4	83.2
CGLF-Swin Transformer	CWRU dataset	98.30	99.0	98.7	98.4
CGLF-CNN	CWRU dataset	94.13	95.3	92.1	89.0
S-Swin Transformer	CWRU dataset	96.20	92.4	91.2	90.0
STFT-SAE	CWRU dataset	93.45	89.5	87.5	85.5
EWT-CNN	CWRU dataset	91.31	92.42	91.31	91.86
WT-BP	CWRU dataset	87.25	85.45	87.25	86.34
S-CNN	Laboratory dataset	86.67	77.6	76.4	75.2
CGLF-Swin Transformer	Laboratory dataset	98.50	99.2	98.9	98.6
CGLF-CNN	Laboratory dataset	93.50	94.2	88.4	83.2
S-Swin Transformer	Laboratory dataset	92.82	93.3	89.1	85.2
STFT-SAE	Laboratory dataset	87.00	78.6	77.3	76.0
EWT-CNN	Laboratory dataset	88.32	85.12	88.32	86.69
WT-BP	Laboratory dataset	84.42	81.45	84.42	82.91

## Data Availability

This data comes from the public data set of Case Western Reserve University, and the link is https://engineering.case.edu/bearingdatacenter/apparatus-and-procedures (accessed on 12 July 2023).

## References

[1] Ren B., Yang M., Chai N., Li Y., Xu D. (2019). Fault Diagnosis of Motor Bearing Based on Speed Signal Kurtosis Spectrum Analysis. Proceedings of the 2019 22nd International Conference on Electrical Machines and Systems (ICEMS).

[2] Ke Z., Di C., Bao X. (2022). Adaptive Suppression of Mode Mixing in CEEMD Based on Genetic Algorithm for Motor Bearing Fault Diagnosis. IEEE Trans. Magn..

[3] Deng W., Zhang S., Zhao H., Yang X. (2018). A Novel Fault Diagnosis Method Based on Integrating Empirical Wavelet Transform and Fuzzy Entropy for Motor Bearing. IEEE Access.

[4] Liao Z., Song X., Jia B., Chen P. (2021). Bearing Fault Feature Enhancement and Diagnosis Based on Statistical Filtering and 1.5-Dimensional Symmetric Difference Analytic Energy Spectrum. IEEE Sens. J..

[5] Kan M.S., Tan A.C.C., Mathew J. (2015). A Review on Prognostic Techniques for Non-Stationary and Non-Linear Rotating Systems. Mech. Syst. Signal Process..

[6] El-Thalji I., Jantunen E. (2015). A Summary of Fault Modelling and Predictive Health Monitoring of Rolling Element Bearings. Mech. Syst. Signal Process..

[7] Yan R., Gao R.X., Chen X. (2014). Wavelets for Fault Diagnosis of Rotary Machines: A Review with Applications. Signal Process..

[8] Rai A., Upadhyay S.H. (2016). A Review on Signal Processing Techniques Utilized in the Fault Diagnosis of Rolling Element Bearings. Tribol. Int..

[9] Wang Y., Xiang J., Markert R., Liang M. (2016). Spectral Kurtosis for Fault Detection, Diagnosis and Prognostics of Rotating Machines: A Review with Applications. Mech. Syst. Signal Process..

[10] Udmale S.S., Singh S.K. (2019). Application of Spectral Kurtosis and Improved Extreme Learning Machine for Bearing Fault Classification. IEEE Trans. Instrum. Meas..

[11] Feng Z., Liang M., Chu F. (2013). Recent Advances in Time–Frequency Analysis Methods for Machinery Fault Diagnosis: A Review with Application Examples. Mech. Syst. Signal Process..

[12] Yu X., Liang Z., Wang Y., Yin H., Liu X., Yu W., Huang Y. (2022). A Wavelet Packet Transform-Based Deep Feature Transfer Learning Method for Bearing Fault Diagnosis under Different Working Conditions. Measurement.

[13] Liu X., Bo L., Luo H. (2015). Bearing Faults Diagnostics Based on Hybrid LS-SVM and EMD Method. Measurement.

[14] Cheng J., Yang Y., Yang Y. (2012). A Rotating Machinery Fault Diagnosis Method Based on Local Mean Decomposition. Digit. Signal Process..

[15] Hu Y., Bao W., Tu X., Li F., Li K. (2020). An Adaptive Spectral Kurtosis Method and Its Application to Fault Detection of Rolling Element Bearings. IEEE Trans. Instrum. Meas..

[16] Frei M.G., Osorio I. (2006). Intrinsic Time-Scale Decomposition: Time–Frequency–Energy Analysis and Real-Time Filtering of Non-Stationary Signals. Proc. R. Soc. A Math. Phys. Eng. Sci..

[17] Zheng J., Cheng J., Yang Y. (2013). A Rolling Bearing Fault Diagnosis Approach Based on LCD and Fuzzy Entropy. Mech. Mach. Theory.

[18] Zheng J., Pan H., Yang S., Cheng J. (2017). Adaptive Parameterless Empirical Wavelet Transform Based Time-Frequency Analysis Method and Its Application to Rotor Rubbing Fault Diagnosis. Signal Process..

[19] Yu K., Lin T.R., Tan J., Ma H. (2019). An Adaptive Sensitive Frequency Band Selection Method for Empirical Wavelet Transform and Its Application in Bearing Fault Diagnosis. Measurement.

[20] Ma P., Zhang H., Fan W., Wang C. (2019). Early Fault Diagnosis of Bearing Based on Frequency Band Extraction and Improved Tunable Q-Factor Wavelet Transform. Measurement.

[21] Guo W. (2020). An Optimal Band-Pass Filter Based on Adaptive Identification of Bearing Resonant Frequency Band. Proceedings of the 2020 Asia-Pacific International Symposium on Advanced Reliability and Maintenance Modeling (APARM).

[22] Wang T., Chu F., Han Q., Kong Y. (2017). Compound Faults Detection in Gearbox via Meshing Resonance and Spectral Kurtosis Methods. J. Sound Vib..

[23] Wang Z., Zhou J., Wang J., Du W., Wang J., Han X., He G. (2019). A Novel Fault Diagnosis Method of Gearbox Based on Maximum Kurtosis Spectral Entropy Deconvolution. IEEE Access.

[24] Sarker I.H. (2021). Deep Learning: A Comprehensive Overview on Techniques, Taxonomy, Applications and Research Directions. SN Comput. Sci..

[25] Yao J., Han T. (2023). Data-Driven Lithium-Ion Batteries Capacity Estimation Based on Deep Transfer Learning Using Partial Segment of Charging/Discharging Data. Energy.

[26] Hoang D.-T., Kang H.-J. (2019). Rolling Element Bearing Fault Diagnosis Using Convolutional Neural Network and Vibration Image. Cogn. Syst. Res..

[27] Sharma C., Kapil S.R., Chapman D. (2021). Person Re-Identification with a Locally Aware Transformer. arXiv.

[28] Liu Z., Lin Y., Cao Y., Hu H., Wei Y., Zhang Z., Lin S., Guo B. (2021). Swin Transformer: Hierarchical Vision Transformer Using Shifted Windows. arXiv.

[29] Li H., Liu T., Wu X., Chen Q. (2020). Application of Optimized Variational Mode Decomposition Based on Kurtosis and Resonance Frequency in Bearing Fault Feature Extraction. Trans. Inst. Meas. Control.

[30] Zhang S., Li L., Liu S., Li J. (2019). Truncation Filtering Method for Envelope Analysis. Int. J. Adapt Control Signal Process.

[31] Wang H., Lai Z., Wu D., Zhang K., Zheng M. (2022). Investigation of the Friction-Induced Vibration of a Novel Four-Way Reversing Valve Using Spectral Kurtosis and Number of Peaks Spectrum. Mech. Syst. Signal Process..

[32] Ma Z., Li X., Liu S., Ge Y., Lu F. (2020). Envelope Demodulation Method Based on SET for Fault Diagnosis of Rolling Bearings under Variable Speed. JAMDSM.

[33] Wang L., Liu Z., Miao Q., Zhang X. (2018). Time–Frequency Analysis Based on Ensemble Local Mean Decomposition and Fast Kurtogram for Rotating Machinery Fault Diagnosis. Mech. Syst. Signal Process..

[34] Yang Z., Cen J., Liu X., Xiong J., Chen H. (2022). Research on Bearing Fault Diagnosis Method Based on Transformer Neural Network. Meas. Sci. Technol..

[35] Tang X., Xu Z., Wang Z. (2022). A Novel Fault Diagnosis Method of Rolling Bearing Based on Integrated Vision Transformer Model. Sensors.

[36] Qin Y., Shi X. (2022). Fault Diagnosis Method for Rolling Bearings Based on Two-Channel CNN under Unbalanced Datasets. Appl. Sci..

[37] Liu H., Li L., Ma J. (2016). Rolling Bearing Fault Diagnosis Based on STFT-Deep Learning and Sound Signals. Shock. Vib..

[38] Hsueh Y.-M., Ittangihal V.R., Wu W.-B., Chang H.-C., Kuo C.-C. (2019). Fault Diagnosis System for Induction Motors by CNN Using Empirical Wavelet Transform. Symmetry.

[39] Zhang J., Sun H., Sun Z., Dong W., Dong Y. (2019). Fault Diagnosis of Wind Turbine Power Converter Considering Wavelet Transform, Feature Analysis, Judgment and BP Neural Network. IEEE Access.

